# Using μPIXE for quantitative mapping of metal concentration in *Arabidopsis thaliana* seeds

**DOI:** 10.3389/fpls.2013.00168

**Published:** 2013-06-03

**Authors:** Magali Schnell Ramos, Hicham Khodja, Viviane Mary, Sébastien Thomine

**Affiliations:** ^1^Institut des Sciences du Végétal, UPR2355, Centre National de la Recherche ScientifiqueGif-sur-Yvette, France; ^2^Chimica Agraria, Dipartimento di Scienze Agrarie e Ambientali, Università degli Studi di UdineUdine, Italy; ^3^Laboratoire d'Etude des Eléments légers, SIS2M, UMR 3299, CEA-CNRS, CEA SaclayGif-sur-Yvette, France

**Keywords:** iron, seed, Arabidopsis, μPIXE, elemental mapping, quantitative

## Abstract

Seeds are a crucial stage in plant life. They contain the nutrients necessary to initiate the development of a new organism. Seeds also represent an important source of nutrient for human beings. Iron (Fe) and zinc (Zn) deficiencies affect over a billion people worldwide. It is therefore important to understand how these essential metals are stored in seeds. In this work, Particle-Induced X-ray Emission with the use of a focused ion beam (μPIXE) has been used to map and quantify essential metals in Arabidopsis seeds. In agreement with Synchrotron radiation X-ray fluorescence (SXRF) imaging and Perls/DAB staining, μPIXE maps confirmed the specific pattern of Fe and Mn localization in the endodermal and subepidermal cell layers in dry seeds, respectively. Moreover, μPIXE allows absolute quantification revealing that the Fe concentration in the endodermal cell layer reaches ~800 μg·g^−1^ dry weight. Nevertheless, this cell layer accounts only for about half of Fe stores in dry seeds. Comparison between Arabidopsis wild type (WT) and mutant seeds impaired in Fe vacuolar storage (*vit1-1*) or release (*nramp3nramp4*) confirmed the strongly altered Fe localization pattern in *vit1-1*, whereas no alteration could be detected in *nramp3nramp4* dry seeds. Imaging of imbibed seeds indicates a dynamic localization of metals as Fe and Zn concentrations increase in the subepidermal cell layer of cotyledons after imbibition. The complementarities between μPIXE and other approaches as well as the importance of being able to quantify the patterns for the interpretation of mutant phenotypes are discussed.

## Introduction

Thanks to their redox properties or Lewis acid strength under physiological conditions, iron (Fe), manganese (Mn), copper (Cu), or zinc (Zn) act as major cofactors in many enzymes such as proteases or antioxidant enzymes as well as in electron transfer chains of mitochondrial respiration or chloroplast photosynthesis (Marschner, 2012). While micronutrients are essential at adequate concentrations, excess leads to toxicity. Moreover, certain trace metals such as cadmium (Cd) or mercury (Hg) have no known function as nutrients and are potentially toxic at low concentrations (Clemens, 2006; Clemens et al., 2013). Therefore, mechanisms for tight regulation of metal homeostasis are vital (Kraemer and Clemens, 2006; Marschner, 2012).

Seeds are a crucial stage in the life of a plant. They contain the macro- and micronutrients necessary to initiate the development of a new organism. More specifically, essential micronutrients need to be safely stored and readily remobilized during early germination. Seeds also represent an important source of nutrient for human beings. Fe and Zn deficiencies affect over a billion people worldwide (Murgia et al., 2012). Consequently, studies on seed transition metal content are necessary. Despite its relatively high abundance, Fe availability remains limited for living organisms. In seeds, Fe is stored either as highly bio-available phytoferritins localized in plastids, or as poorly bio-available Fe-phytate salts, localized in vacuolar inclusions called globoids (Harrison and Arosio, 1996; Briat and Lobreaux, 1997; Otegui et al., 2002; Lanquar et al., 2005). The balance between those two forms is different according to plant species and is greatly modified during germination. Besides, in seeds, phytate salts are also the main storage form of potassium (K), magnesium (Mg), calcium (Ca), and Zn (Mikus et al., 1992).

In Arabidopsis, VIT1, a vacuolar Fe transporter required for Fe storage in the vacuole during seed formation, was identified by Kim et al. (2006). Synchrotron radiation X-ray fluorescence (SXRF) tomographic imaging demonstrated that loss of VIT1 function disrupts the cell specific localization of Fe in dry seeds. Whereas Fe is stored in endodermal cells surrounding the provascular tissues in wild type (WT), it co-localizes with Mn in the subepidermal cell layer of *vit1-1* knockout mutant embryos (Kim et al., 2006; Roschzttardtz et al., 2009). Fe mislocalization results in drastically decreased viability of *vit1-1* seedlings under Fe deficiency. While VIT1 mediates Fe influx into the vacuole, NRAMP3 and NRAMP4 metal transporters have been shown to act redundantly to export Fe out of the vacuole (Lanquar et al., 2005). Energy-Dispersive X-ray (EDX) technique indicated that the *nramp3nramp4* double knockout mutant is defective in Fe retrieval from seed vacuolar globoids during germination. As a consequence, *nramp3nramp4* mutant seedlings display an early developmental arrest when germinated on low Fe. Furthermore, the drastic morphological and biological changes that occur during germination must be accompanied by a relocation of nutrients to the sites where they are required for metabolism. Although several reports have addressed metal patterning in dry seed, the changes in metal localization and their kinetics upon seed germination have not been addressed at the tissue level. Three imaging techniques have been used to investigate Fe distribution in Arabidopsis WT and mutant seeds EDX (Lanquar et al., 2005), SXRF (Kim et al., 2006), and Perls/DAB staining (Roschzttardtz et al., 2009). However, those techniques provided either non-quantitative data (EDX, Perls/DAB) or approximate quantification (SXRF) of metal concentrations in the different seed tissues.

Particle-Induced X-ray Emission induced by a focused ion beam (μPIXE) allows multi-elemental mapping in biological samples with high spatial resolution (1 μm range) and high sensitivity (down to μg·g^−1^ range). Importantly, μPIXE technique presents the unique advantage of providing quantitative results when used simultaneously with Rutherford Backscattering (RBS) and Scanning Transmission Ion Microscopy (STIM) analyses (Deves et al., 2005). The combined measurements of trace element amount by PIXE, charge monitoring and organic element determination by RBS and sample local mass determination by STIM are often referred as “fully” quantitative results in the literature in opposition to “semi”-quantitative results obtained by other imaging techniques.

In plants, μPIXE has been used for the localization and quantification of essential macro- and micronutrients in specific tissues and organs such as elemental mapping of buckwheat seeds (Vogel-Mikus et al., 2009), Fe in barley roots (Schneider et al., 2002), Fe and Zn in Phaseolus seeds (Cvitanich et al., 2010, 2011), Cu in *Brassica carinata* leaf and root (Cestone et al., 2012). μPIXE was also used to image and quantify non-essential elements. In the context of environmental contamination, μPIXE was used to study cesium (Cs) in Arabidopsis leaf, stem, and trichome (Isaure et al., 2006), Cd and Ni in soybean seed (Malan et al., 2012), and uranium (U) in leaf and root of oilseed rape, sunflower, and wheat (Laurette et al., 2012). In the context of metal hyperaccumulation, it was used to analyze Cd in leaf and seed of *Thalspi praecox* (Vogel-Mikus et al., 2007, 2008) or nickel (Ni) in *Berkheya coddii* leaves (Budka et al., 2005).

Here, the μPIXE approach was used to quantitatively analyse metal distribution in Arabidopsis seeds. A sample preparation protocol suitable for μPIXE analysis of *Arabidopsis thaliana* dry but also imbibed seeds was established. Analysis of some nutritionally important elements by μPIXE mapping confirmed the previously established pattern in WT dry seed metal distribution, exhibiting Mn accumulation at the abaxial side of cotyledons as well as Fe localization around the provascular tissues. Local Fe, Mn, and Zn concentrations were determined in these tissues in WT and both *nramp3nramp4* and *vit1-1* mutant seeds. Moreover, a comparison between elemental maps obtained with dry or imbibed seeds revealed early changes in metal localization. The μPIXE results are put in perspective with other elemental analysis techniques raising questions regarding the input of element quantification in the interpretation of mutant phenotypes. Finally, μPIXE results obtained for WT dry and imbibed seeds are discussed with respect to the possible use of this technique to study dynamic element redistribution.

## Materials and methods

### Plant material

Mature (dry) and imbibed seeds of Arabidopsis (*Arabidopsis thaliana* accession Columbia-0) WT, *nramp3nramp4* (*nr3nr4*) and *vit1-1* mutants were used. Generation of both mutants has been described previously in Ravet et al. (2009b) and Kim et al. (2006), respectively. Dry seeds of all genotypes were harvested from plants grown on potting soil (Tonerde PAM argile, Brill France) in a greenhouse under a 16 h photoperiod with regular watering (2–3 times per week). Seeds were imbibed in deionized water on an orbital shaker (40 rotations·min^−1^), under continuous light (60 μmol photon·m^−2^·s^−1^) at 21°C for 48 h.

### AAS analyses of dry seeds

Three to four replicates of circa 20 mg of dry seeds were digested in 2 ml of 70% nitric acid in a DigiBlock ED36 (LabTech, Italy) at 80°C for 1 h, 100°C for 1 h, and 120°C for 2 h. After dilution to 12 ml with ultrapure water, K, Ca, Mn, Fe, Cu, and Zn contents of the samples were determined by atomic absorption spectrometry using an AA240FS flame spectrometer (Agilent, USA).

### Sample preparation for μPIXE analysis

In order to avoid losses of elements and their redistribution in the analysed samples, cryotechniques were used. The seeds were fixed by high pressure cryofixation (Leica EM PACT2, Leica Microsystems, Germany) and stored in liquid nitrogen. Subsequent steps were performed in a cryostat (CM3050-S, Leica Microsystems, Germany) at −30°C: seeds were recovered from their cryofixation container in cold isopentane, embedded in an inert matrix (Tissue-Tek® OCT compound, Sakura Finetek, USA) and cut with a tungsten blade in sections of approximately 30 μm. Sections were individually mounted on a cold aluminium sample holder covered with a pioloform film (1 g pioloform, 75 ml chloroform). Samples were carefully transferred to a freeze-dryer (Alpha 1-4, Christ Martin, Germany), freeze-dried at −10°C, under a vacuum of 0.37 mbar for 48 h and brought back to room temperature (RT) by steps of 5°C per hour. Finally, samples were covered by a second pioloform film and stored in an anhydrous environment at RT until μPIXE analyses. Section integrity of seed sample was verified by putting a few freshly cut sections directly on a microscope slide (Menzel-Gläser SUPERFROST®, Thermo Fisher Scientific, USA) stored in the cryostat. After sectioning, the microscope slides mounted with seed sections were quickly brought to RT in a vacuum desiccator and checked under a light microscope.

### Multi-elemental localization by μPIXE

Microanalyses of Arabidopsis sections were performed at the Saclay nuclear microprobe, France (Khodja et al., 2001). The sample holder with seed sections and reference samples were attached to a motorized vacuum goniometer in the analyzing chamber. After closing the chamber and establishing a vacuum (typically 10^−6^ mbar), rough positioning of the samples was achieved using an optical camera. The proton beam of 3.0 MeV and 200 pA current was focused down to a 1.5 × 1.5 μm^2^ spot, used to rapidly scan large areas of the sample and finally adjusted to scan the seed section areas (usually 500 × 500 μm^2^) during circa 4 h. Simultaneous PIXE, RBS, and STIM analyses were performed. A 40 mm^2^-Bruker XFlash SDD detector placed at 75° relative to the beam path and at 21 mm working distance of the scanned sample was used to collect X-rays emitted by non-organic elements. A 50 μm Mylar foil was placed in front of the detector to stop backscattered protons and attenuate X-ray signals from major elements. Backscattered particles were collected by a 180 mm^2^-surface barrier detector positioned at 170° and 35 mm from incident beam. Energy-loss maps based on STIM were obtained by collecting scattered particles at 30° using a surface barrier detector located behind the sample. In total, 11 seed sections were analyzed: 5 from WT, 5 from *nramp3nramp4* mutant and 1 from *vit1-1* mutant.

### Multi-elemental quantification by μPIXE

In order to generate μPIXE qualitative elemental images, PIXE spectra of the scanned Arabidopsis sections were processed using RISMIN software (Daudin et al., 2003). Distinct regions of particular interest (ROIs) such as seed morphological structures or areas enriched for one particular element were selected as ROIs on the elemental images and the corresponding X-ray spectra were extracted. Associated RBS and STIM spectra and images were used to assess thickness and matrix composition of the different ROIs using SIMNRA (Mayer, 1999) and a modified version of RISMIN software. Finally, all elemental quantifications were obtained by processing the extracted data using SIMNRA and GUPIX (Campbell et al., 2000) softwares. Concentrations of the elements present in the selected ROIs (whole seed section, Fe- and Mn-enriched areas) are presented, either as elemental concentrations of one representative sample (Table 2), or as average elemental concentrations of three to four representative samples (Figures 5A,B).

### Data analyses

Average elemental concentrations presented in (Figures 5A,B) as well as elemental fractions presented in Table 3, were analysed by a Kruskal–Wallis test followed by a Tukey *post-hoc* test for multiple comparisons (*p* < 0.05). For both dry and imbibed seeds, fractions of Fe and Mn that accumulate in their respective enriched areas compared to the whole seed section element content were calculated as followed: [element concentration in enriched area·area of enriched area]/[element concentration in whole seed section·area of whole seed section].

## Results

### Sample preparation for analyses

**Figure 1:**
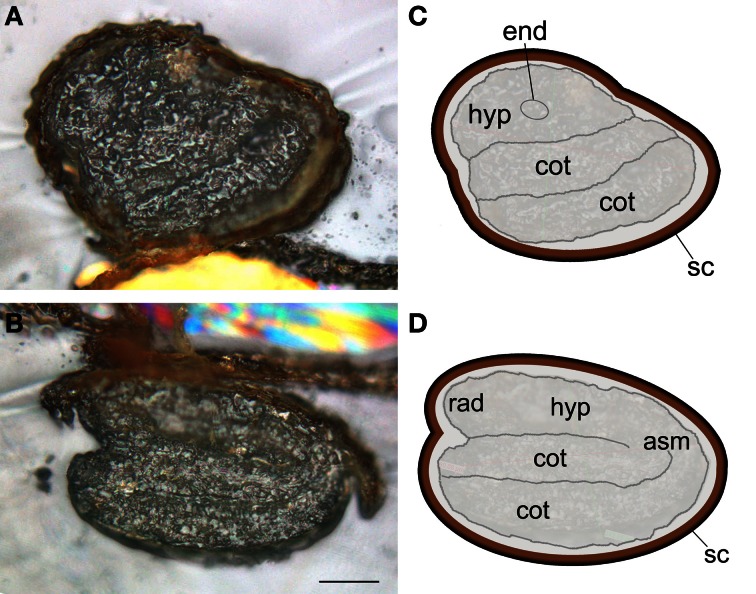
***Arabidopsis thaliana* dry seed structure**. Light microscopy images of transversal **(A)** and longitudinal **(B)** sections with respective schematic representations **(C,D)**. Thirty micrometer sections were obtained at −30°C using a cryomicrotome. Scale bar: 100 μm; asm, apical shoot meristem; cot, cotyledon; end, endodermis; hyp, hypocotyl; rad, radicle; sc, seed coat.

Seed samples were prepared as recommended in Mesjasz-Przybylowicz and Przybylowicz (2002) and described in Materials and Methods. The chosen section thickness was 30 μm to obtain the best compromise between 3 requirements: (1) analysing a sufficient amount of matter by μPIXE to obtain accurate quantification, (2) the ability to record RBS and STIM complementary signals, and (3) to resolve Arabidopsis seed morphology (30 μm corresponds to circa 2 cell layers). A cryogenic approach was used to prevent element leakage or redistribution within the sample sections (Mesjasz-Przybylowicz and Przybylowicz, 2002). Due to Arabidopsis seed small size, high-pressure freezing was used. In order to confirm that cryosectioning did not alter seed structure, some sections were put aside and directly observed under light microscopy after being rapidly brought back to RT. In agreement with previous reports, Arabidopsis dry seed morphological structure could be clearly defined for 30 μm thick longitudinal and transversal sections (Figure 1) (e.g., Vaughan and Whitehouse, 1971). After high-pressure freezing, samples could be either freeze-dried or freeze-substituted as no module for frozen-hydrated state specimen analysis was available (Tylko et al., 2007). Freeze-drying technique has been demonstrated to be neither suitable for observations at the cellular level since it may alter the morphology of the samples, nor for metal speciation since water extraction induces metal speciation artifacts (Sarret et al., 2009). However, freeze-drying is appropriate for elemental mapping, in particular for Fe, at the tissue level and thus, was used in this research (Schneider et al., 2002; Sarret et al., 2013). Furthermore, preliminary tests showed that the substitution of vitrified water by EPOXY resin lead to partial to complete leakage of some elements (data not shown).

### Arabidopsis seed elemental distribution using μPIXE technique

**Figure 2:**
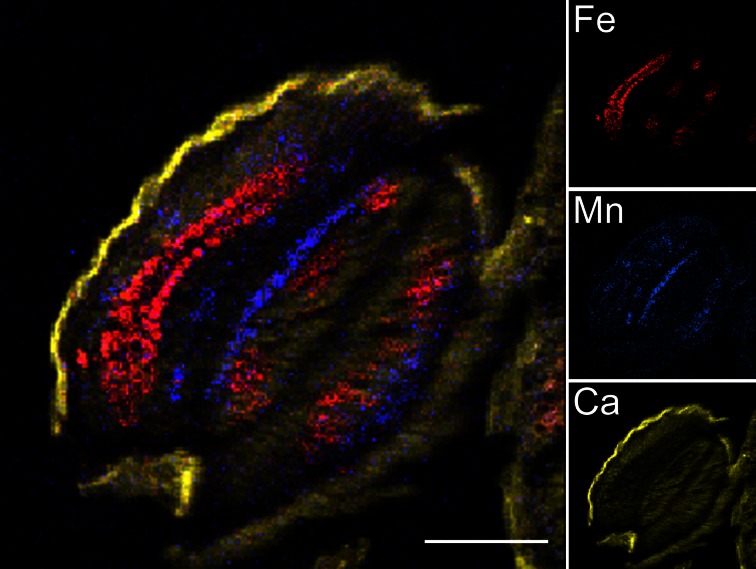
μ**PIXE elemental reconstituted image of *Arabidopsis thaliana* dry seed section**. Total X-ray spectra of areas scanned with 3 MeV proton beam were collected. Specific distributions of Fe, Mn, and Ca in a 30 μm thick longitudinal section of Arabidopsis dry seed are shown overlaid (large image) and separately (small images). False color images and reconstitution were obtained using ImageJ free software. Scale bar: 100 μm; red, Fe; blue, Mn; yellow, Ca.

**Figure 3:**
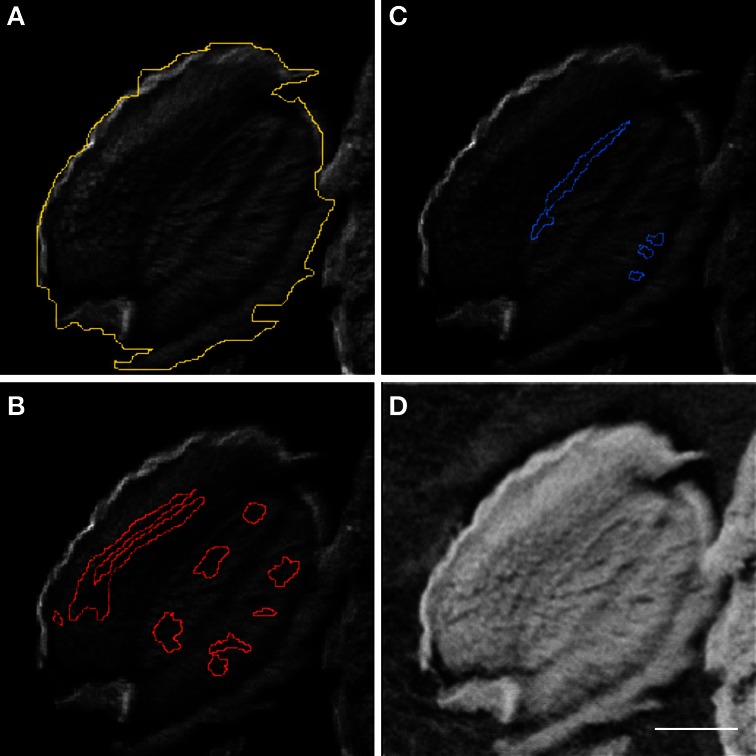
**Regions of interest for μPIXE elemental quantification**. Element distribution maps allowed selection of regions of interest (ROI). *Arabidopsis thaliana* whole seed **(A)**, Fe- **(B)**, and Mn- **(C)** ROIs are shown using the Ca distribution map as background. The associated STIM image **(D)** shows the local mass of the sample which is necessary for quantification. Scale bar: 100 μm.

**Figure A1:**
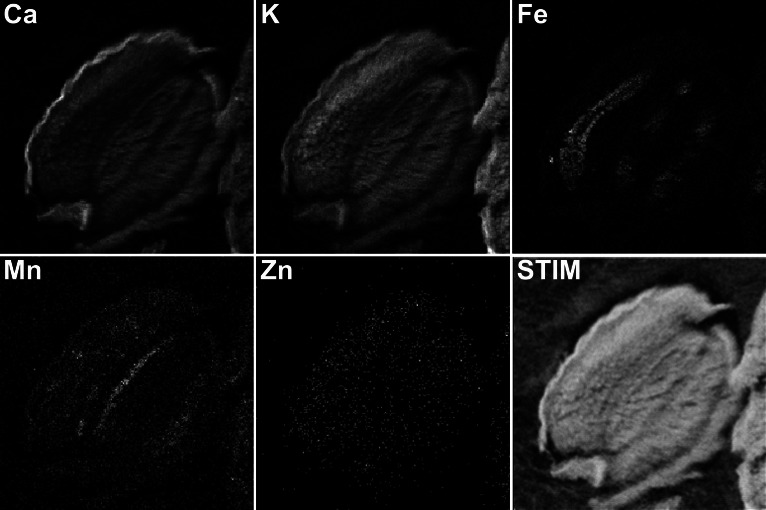
**μPIXE elemental reconstituted image of *Arabidopsis thaliana* dry seed section**. Total X-ray spectra of areas scanned with 3 MeV proton beam were collected. Specific distributions of Ca, K, Fe, Mn, and Zn in a 30 μm thick longitudinal section of Arabidopsis dry seed are separately. The associated STIM image shows the local mass of the sample. Scale bar: 100 μm.

Freeze-dried 30 μm thick sections of *Arabidopsis thaliana* dry and imbibed seeds were analysed by Particle-Induced X-ray Emission induced by a focused ion beam (μPIXE) and elemental images were generated (Figure 2). Some macroelements, such as K, and microelements such as Zn are apparently evenly distributed over the dry seed section (Figure A1). Mapping of Ca, which is evenly distributed in the embryo and accumulates to a higher level in the seed coat, allows the visualization of general seed morphology, recalling the light microscopy image (compare Figures 1B, 2, Ca inset). Other elements, such as Fe and Mn, exhibited more specific localization in particular structures, organs or tissues. The elemental map of Fe highlighted the provascular tissue in the cotyledons, hypocotyl and radicle while the Mn elemental map showed concentration on the abaxial (lower) side of the cotyledons (Figure 2, Fe and Mn insets). Subsequently, elemental images were used to select regions specifically enriched in Fe or Mn within sections (regions of interest, ROIs; Figure 3) to determine the element concentrations within ROIs.

### Seed elemental content analyses

Dry seed bulk sample analyses of Arabidopsis WT, *nramp3nramp4* and *vit1-1* were performed by atomic absorption spectroscopy (AAS) to determine seed concentrations of various macro- and microelements (Table 1). As previously reported, for all quantified elements, no notable differences were observed between seeds of the different genotypes (Lanquar et al., 2005; Kim et al., 2006; Young et al., 2006). Some seeds of the analysed batches were set aside and analysed by μPIXE to determine elemental concentration in the ROI corresponding to the whole seed section (Table 1). In general, seed element average concentrations obtained by μPIXE for each genotype were in the same range of concentrations but usually higher when compared to AAS analyses (Table 1). This difference can be partly explained by the huge difference in the sample sizes used for each technique. AAS elemental content analyses of circa 1800 Arabidopsis dry seeds (20 mg aliquots) are compared with μPIXE analyses of few 30 μm-thick seed sections (approximately 2 cell layers). Single seed elemental variability, seed cell heterogeneity as well as low replicate number inherent to the technique have a larger impact on the seed elemental content measured by μPIXE.

**Table 1:** **AAS analysis and μPIXE quantification of various elements in three different *Arabidopsis thaliana* seed genotypes**.

**AAS—dry seed**					**μPIXE—seed section**					
**El.**	**WT**	***nr3nr4***	***vit1-1***		**WT**		***nr3nr4***		***vit1-1***	
	μ**g · g^−1^_DW_**	μ**g · g^−1^_DW_**	μ**g · g^−1^_DW_**	**LOD**	μ**g · g^−1^_DW_**	**LOD**	μ**g · g^−1^_DW_**	**LOD**	μ**g · g^−1^_DW_**	**LOD**
K	11881 ± 188	11272 ± 463	11909 ± 663	0.008	17648	36	19989	30	27274	52
Ca	4200 ± 208	4590 ± 288	4445 ± 329	0.001	9165	85	11223	82	9757	77
Mn	32 ± 4	34 ± 3	33 ± 3	0.001	44	4	58	4	65	8
Fe	78 ± 5	73 ± 1	80 ± 13	0.006	159	3	189	3	157	8
Cu	10 ± 1	12 ± 1	11 ± 0	0.001	10	2	20	2	68	3
Zn	58 ± 5	59 ± 7	62 ± 5	0.001	141	2	197	2	169	6

*AAS of bulk samples. Values are means ± SE (n = 3–4 dry seed batches for each genotypes). X-ray and RBS spectra of the whole seed section were selected using RISMIN software and analysed using SIMNRA and GUPIX softwares. Concentrations of one representative measured sample are presented; LOD, limit of detection of the measurements (μg·g^−1^_DW_)*.

**Figure 4:**
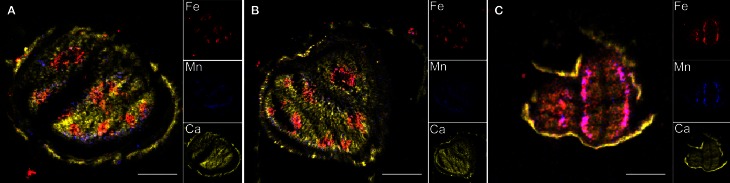
**Transversal sections of *Arabidopsis thaliana* dry and imbibed seeds**. Specific distributions of Fe, Mn, and Ca in section of WT **(A)** and *nr3nr4* **(B)** imbibed seeds and *vit1-1* **(C)** dry seeds are shown overlaid (large image) and separately (small images). Scale bar: 100 μm; red, Fe; blue, Mn; yellow, Ca.

Elemental images obtained for the three genotypes showed that Fe distribution is strongly perturbed in *vit1-1* mutant whereas it is not affected in *nramp3nramp4* dry seed at the same stage (Figure 4). Similar elemental distribution patterns were obtained using SXRF microtomography (Kim et al., 2006; Donner et al., 2012). ROIs corresponding to whole seed section, Fe- or Mn-enriched areas were selected on the images of each genotype and the corresponding PIXE, RBS, and STIM extracted spectra were analysed (Table 2). In WT and *nramp3nramp4* dry seed sections, element quantification within the ROIs confirmed the accumulation of Fe around the provascular tissues of the radicle and cotyledons (from 159 μg·g^−1^ dry weight (DW) in the whole section, up to 751 μg·g^−1^_DW_ in Fe-enriched area) and of Mn at the abaxial side of cotyledons (from 44 μg·g^−1^_DW_ up to 386 μg·g^−1^_DW_). Surprisingly, slight accumulation of Zn also occurred in the Fe-enriched area (from 141 μg·g^−1^_DW_ up to 311 μg·g^−1^_DW_). In *vit1-1* dry seed, Fe (up to 360 μg·g^−1^_DW_) and Mn (up to 172 μg·g^−1^_DW_) concentrated in a single Fe- and Mn-enriched area at the abaxial side of the cotyledons, while no Fe accumulation around provascular tissues was observed. Here also, Zn (up to 224 μg·g^−1^_DW_) exhibited a slight accumulation within the combined enriched area.

**Table 2:** **μPIXE quantification of various elements in three different *Arabidopsis thaliana* seed genotypes with emphasis on Fe- and Mn-enriched areas**.

**El.**	**Seed section**		**Fe-enriched area**		**Mn-enriched area**	
	μ**g · g^−1^_DW_**	**LOD**	μ**g · g^−1^_DW_**	**LOD**	μ**g · g^−1^_DW_**	**LOD**
**WT**						
K	17648	36	21393	71	15851	121
Ca	9165	85	7509	140	9060	155
Mn	44	4	22	12	386	13
Fe	159	3	751	5	90	20
Cu	10	2	9	7	n.d.	n.d.
Zn	141	2	311	11	156	15
***nr3nr4***						
K	19989	30	19443	81	15582	57
Ca	11223	82	10672	128	10315	90
Mn	58	4	85	13	106	7
Fe	189	3	520	11	218	6
Cu	20	2	24	7	9	5
Zn	197	2	281	11	200	5
**El.**	**Seed section**		**Fe- and Mn-enriched area**			
	μ**g · g^−1^_DW_**	**LOD**	**μg · g^−1^_DW_**	**LOD**		
***vit1-1***						
K	27274	25	22519	52		
Ca	9757	63	8826	77		
Mn	65	3	172	8		
Fe	157	3	360	8		
Cu	68	2	60	3		
Zn	169	1	224	6		

*Concentrations of one representative measured sample of dry seed are presented; LOD, limit of detection of the measurements (μg·g^−1^_DW_); n.d., not detected*.

**Figure 5:**
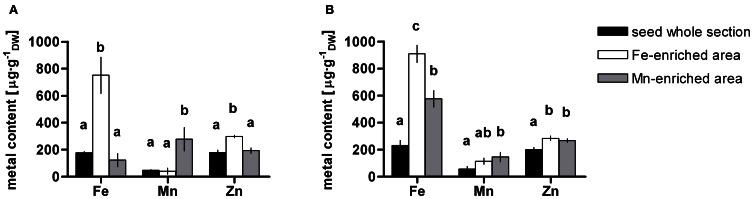
**Metal content in *Arabidopsis thaliana* dry **(A)** and imbibed **(B)** seed**. Fe, Mn, and Zn contents in whole seed section (black bars) as well as in selected Fe- (white bars) and Mn- (gray bars) enriched areas were quantified. Dry seeds were cryofixed directly **(A)** or after 48 h imbibition **(B)**. Values are means ± SE (*n* = 3–4 scanned sections). Different letters denote statistically significant differences based on Kruskal–Wallis test followed by Tukey *post-hoc* test comparing values of dry and imbibed seeds for each metal (*P* < 0.05).

The concentrations of Fe, Mn, and Zn in whole longitudinal and transversal seed sections as well as Fe- and Mn-enriched areas from dry and imbibed samples of WT and *nramp3nramp4* were measured. Statistical analyses of the results did not reveal any significant difference between WT and *nramp3nramp4* in any of the ROIs analysed. Figure 5 shows the average concentrations of Fe, Mn, and Zn in the whole seed section, Fe- and Mn-enriched areas from dry and imbibed samples. In both sample types, Fe (751 ± 134 μg·g^−1^_DW_ and 910 ± 74 μg·g^−1^_DW_) significantly accumulated in Fe-enriched provascular area as expected (Figures 5A,B). As already indicated in Table 2, Zn average concentrations were also significantly higher in Fe-enriched areas of both dry and imbibed seeds (298 ± 9 μg·g^−1^_DW_ and 284 ± 18 μg·g^−1^_DW_). Mn accumulated at significantly higher concentration in the Mn-enriched area at the abaxial side of the cotyledons of both dry and imbibed seeds (Figures 2, 4, 5 and Table 2). Interestingly, after 48 h of imbibition, Fe (577 ± 61 μg·g^−1^_DW_) and Zn (268 ± 14 μg·g^−1^_DW_) were also significantly more concentrated within Mn-enriched area at the abaxial side of the cotyledons. These changes were not detectable visually on the elemental maps.

Finally, combining the relative areas of Fe- and Mn-enriched areas and the metal concentration within these areas, we calculated the fractions of Fe and Mn accumulated in their corresponding enriched areas, namely the provascular tissue region for Fe and the abaxial side of the cotyledons for Mn (Table 3). These calculations revealed that at most 69 and 54% of Fe and Mn, respectively, were localized within these areas. A significant fraction of seed Fe and Mn stores is thus localized outside the areas where these metals are the most concentrated.

**Table 3:** **Fractions of Fe and Mn in *Arabidopsis thaliana* dry and imbibed seed**.

**El.**	**Dry**		**Imbibed**	
	**% metal**	**% area**	**% metal**	**% area**
**Fe**	50.7 ± 11.2	13.4 ± 5.4	68.9 ± 5.8	17.1 ± 1.5
**Mn**	30.3 ± 7.0	8.3 ± 6.0	54.4 ± 2.8^*^	22.4 ± 7.3

*The fractions of Fe and Mn quantified in their corresponding enriched areas relative to the whole seed section element content were calculated for dry and imbibed seed. Proportion of each enriched area was also calculated. Values are means ± SE (n = 3–4 scanned sections). Asterisk denotes statistically significant differences based on Mann–Whitney U test (p < 0.05)*.

## Discussion

Images of Arabidopsis dry seed sections obtained by μPIXE confirmed Fe, Mn, and Zn distribution patterns observed previously in WT, *nramp3nramp4* and *vit1-1* mutant dry seeds. In addition, valuable information about local metal concentrations could be obtained from the analyses of the different types of spectra collected at the microprobe installation.

### μPIXE complements other techniques for elemental imaging of seeds

Fe distribution in Arabidopsis seeds has been already reported using different imaging techniques: EDX microanalysis on a transmission electron microscope (Lanquar et al., 2005), Fe histochemical localization using Perls staining with DAB enhancing (Roschzttardtz et al., 2009) and SXRF microtomography (Kim et al., 2006; Chu et al., 2010; Donner et al., 2012). The respective advantages and limitations of these different approaches have been recently discussed in several reviews (Deves et al., 2005; Lobinski et al., 2006; Ortega et al., 2009; Punshon et al., 2009, 2012; Donner et al., 2012; Sarret et al., 2013).

Combined together, the information about Fe localization obtained by EDX, histochemical staining and SXRF techniques indicate that in Arabidopsis dry seed, Fe is concentrated in the endodermal cells of the embryo and, at subcellular level, is associated with vacuolar globoids (Lanquar et al., 2005; Kim et al., 2006; Roschzttardtz et al., 2009). Fe concentrations close to 1000 μg·g^−1^_DW_ in provascular tissues were quantified by μPIXE (Table 2 and Figure 5). As globoids represent no more than 10% of the cell volume but constitute the main site for Fe storage in seed cells (Lanquar et al., 2005), endodermal vacuole globoids possibly contain close to 1% Fe. Besides, it has been hypothesized that Fe in the vacuole is mainly stored as Fe^3+^ but this still remains to be demonstrated using XAS (Pich et al., 2001; Otegui et al., 2002). Based on analyses of mutant phenotypes and metal localization, a mechanism of Fe loading into seed endodermal vacuoles mediated by VIT1 and its release into vasculature cells by NRAMP3 and NRAMP4 during germination has been suggested (Morrissey and Guerinot, 2009; Roschzttardtz et al., 2009). Accordingly, recent data indicated that no more than 5% of total seed Fe is associated with the plastidial ferritin in Arabidopsis seed (Ravet et al., 2009a). Concentration of Fe in endodermal vacuoles in the vicinity of the provascular strand cells allows rapid and easy mobilization of Fe to the growing parts of the seedling during germination. Nevertheless, our determination of Fe fractions in dry seed sections by μPIXE revealed that only about 50% of this metal is actually stored around the seed embryo provascular tissue (Table 3). This result indicates that Arabidopsis seeds likely contain two pools of Fe: a pool of concentrated Fe associated to endodermal vacuoles, previously identified by several imaging techniques (Kim et al., 2006; Roschzttardtz et al., 2009), and a less concentrated pool spread in all seed tissues, which have so far been overseen. This finding does not contradict the previous assessments about the central role of vacuoles in Fe storage in Arabidopsis (Roschzttardtz et al., 2009). However, it points to the existence of significant Fe stores outside of the endodermal cells that have to be taken into account in seed Fe storage models. Moreover, our results suggest that, besides VIT1, which is expressed near the vasculature, other vacuolar Fe uptake systems drive Fe accumulation into the vacuoles of other embryo cells.

Imaging techniques also showed heterogeneous distribution of Mn and Zn in plant seeds (Kim et al., 2006; Vogel-Mikus et al., 2007; Takahashi et al., 2009; Cvitanich et al., 2011). Our analyses by μPIXE showed that up to 30% of the Mn present in Arabidopsis dry seed is stored in subepidermal cells. Until now, the transporters which mediate Mn accumulation in those particular cells remain unknown. In the case of Zn, our quantification of Zn in seed sections by μPIXE indicated that Zn is slightly more concentrated in the embryo provascular tissue. This is in agreement with SXRF microtomography of Arabidopsis seeds (Kim et al., 2006) and μPIXE analysis of Phaseolus seeds (Cvitanich et al., 2011).

### Metal redistribution in arabidopsis seed during germination monitored by μPIXE

During germination, seed undergoes drastic morphological and biological changes, necessarily inducing a redistribution of macro- and micronutrients (Bewley, 1997; Bolte et al., 2011). Whereas mature dry seed is an excellent plant sample stage for imaging by SXRF or μPIXE due to its natural dehydration state, monitoring of seed germination by X-ray imaging techniques remains challenging. Here, a μPIXE analysis of Arabidopsis WT and *nramp3nramp4* mutant after seed imbibition was undertaken. Whereas EDX microanalyses revealed *nramp3nramp4* mutant defect in Fe remobilization from globoids in 2-day old seedling, μPIXE failed to detect any qualitative or quantitative modification of the Fe pattern between the two genotypes at the same stage (Lanquar et al., 2005). As a matter of fact, elemental maps obtained on dry seeds or after imbibitions did not show any obvious difference. Two hypotheses may account for this apparent discrepancy: (1) even though the experiment was performed after 2 days in both case, seedling development may have been slower in the case of the μPIXE experiment, due to differences in the conditions or seed age. Attempts to analyse sections of WT and mutant seeds at later stage were hampered by difficulties to obtain intact sections; (2) after 2 days, Fe may have been remobilized from the globoids, preventing its detection by EDX, but may have remained in the same tissues explaining why no change could be detected at the μm resolution of the μPIXE analysis.

Nevertheless, quantification of Fe, Mn, and Zn in seed sections by μPIXE indicates significant distribution modifications after 48 h of imbibition. During imbibition, Fe and Zn concentrations increase in subepidermal cells of cotyledons, corresponding to the Mn-enriched area, while the fraction of Mn accumulating in those cells significantly increases from 30 to more than 50% of total Mn content. Although the origin of the metals could not be identified as no significant decrease of Fe, Mn, or Zn occurs in other areas, it is likely that during seed imbibition metals are already beginning to be redistributed toward the future photosynthetic cell layers. As the Fe concentration in the endodermal area is unchanged after imbibition, it is tempting to speculate that the Fe that accumulates in the sub epidermal area originates from the “diluted pool” spread in all seed tissues, which would therefore represent a more loosely bound Fe pool. The endodermal pool would be released at later stages of germination.

In conclusion, our study using μPIXE complemented other approaches to analyse metal distribution in Arabidopsis seeds. This fully quantitative approach revealed unsuspected important Fe stores outside the endodermal cells. Analysis after seed imbibition provided a first glimpse at the rapid metal redistribution that occurs during germination.

### Conflict of interest statement

The authors declare that the research was conducted in the absence of any commercial or financial relationships that could be construed as a potential conflict of interest.

## References

[B1] Bewley J. D. (1997). Seed germination and dormancy. Plant Cell 9, 1055–1066 10.1105/tpc.9.7.1055 12237375 PMC156979

[B2] Bolte S. Lanquar V. Soler M. N. Beebo A. Satiat-Jeunemaitre B. Bouhidel K. (2011). Distinct lytic vacuolar compartments are embedded inside the protein storage vacuole of dry and germinating *Arabidopsis thaliana* seeds. Plant Cell Physiol. 52, 1142–1152 10.1093/pcp/pcr065 21613277

[B3] Briat J. F. Lobreaux S. (1997). Iron transport and storage in plants. Trends Plant Sci. 2, 187–193 10.1016/S1360-1385(97)85225-9

[B4] Budka D. Mesjasz-Przybylowicz J. Tylko G. Przybylowicz W. J. (2005). Freeze-substitution methods for Ni localization and quantitative analysis in *Berkheya coddii* leaves by means of PIXE. Nucl. Instrum. Methods Phys. Res. B 231, 338–344 10.1016/j.nimb.2005.01.080

[B5] Campbell J. L. Hopman T. L. Maxwell J. A. Nejedly Z. (2000). Guelph PIXE software package III: alternative proton database Nucl. Instrum. Methods Phys. Res. B 170, 193–204 10.1016/S0168-583X(00)00156-7

[B6] Cestone B. Vogel-Mikus K. Quartacci M. F. Rascio N. Pongrac P. Pelicon P. (2012). Use of micro-PIXE to determine spatial distributions of copper in Brassica carinata plants exposed to CuSO 4 or CuEDDS. Sci. Total Environ. 427–428, 339–346 10.1016/j.scitotenv.2012.03.065 22542302

[B6a] Chu H. H. Chiecko J. Punshon T. Lanzirotti A. Lahner B. Salt D. E. (2010). Successful reproduction requires the function of Arabidopsis YELLOW STRIPE-LIKE1 and YELLOW STRIPE-LIKE3 metal-nicotianamine transporters in both vegetative and reproductive structures. Plant Physiol. 154, 197–210 10.1104/pp.110.159103 20625001 PMC2938154

[B7] Clemens S. (2006). Toxic metal accumulation, responses to exposure and mechanisms of tolerance in plants. Biochimie 88, 1707–1719 10.1016/j.biochi.2006.07.003 16914250

[B8] Clemens S. Aarts M. G. M. Thomine S. Verbruggen N. (2013). Plant science: the key to preventing slow cadmium poisoning. Trends Plant Sci. 18, 92–99 10.1016/j.tplants.2012.08.003 22981394

[B9] Cvitanich C. Przybylowicz W. J. Mesjasz-Przybylowicz J. Blair M. W. Astudillo C. Orlowska E. (2011). Micro-PIXE investigation of bean seeds to assist micronutrient biofortification. Nucl. Instrum. Methods Phys. Res. B 269, 2297–2302 10.1016/j.nimb.2011.02.047

[B10] Cvitanich C. Przybylowicz W. J. Urbanski D. F. Jurkiewicz A. M. Mesjasz-Przybylowicz J. Blair M. W. (2010). Iron and ferritin accumulate in separate cellular locations in Phaseolus seeds. BMC Plant Biol. 10:26 10.1186/1471-2229-10-26 20149228 PMC2831038

[B11] Daudin L. Khodja H. Gallien J. P. (2003). Development of “position-charge-time” tagged spectrometry for ion beam microanalysis. Nucl. Instrum. Methods Phys. Res. B 210, 153–158 10.1016/S0168-583X(03)01008-5

[B12] Deves G. Isaure M. P. Le Lay P. Bourguignon J. Ortega R. (2005). Fully quantitative imaging of chemical elements in *Arabidopsis thaliana* tissues using STIM, PIXE and RBS. Nucl. Instrum. Methods Phys. Res. B 231, 117–122 10.1016/j.nimb.2005.01.044

[B13] Donner E. Punshon T. Guerinot M. L. Lombi E. (2012). Functional characterisation of metal(loid) processes in planta through the integration of synchrotron techniques and plant molecular biology. Anal. Bioanal. Chem. 402, 3287–3298 10.1007/s00216-011-5624-9 22200921 PMC3913160

[B14] Harrison P. M. Arosio P. (1996). The ferritins: molecular properties, iron storage function and cellular regulation. Biochim. Biophys. Acta Bioenerg. 1275, 161–203 10.1016/j.jsb.2008.12.001 8695634

[B15] Isaure M. P. Fraysse A. Deves G. Le Lay P. Fayard B. Susini J. (2006). Micro-chemical imaging of cesium distribution in *Arabidopsis thaliana* plant and its interaction with potassium and essential trace elements. Biochimie 88, 1583–1590 10.1016/j.biochi.2006.08.006 16987577

[B16] Khodja H. Berthoumieux E. Daudin L. Gallien J. P. (2001). The Pierre Süe Laboratory nuclear microprobe as a multi-disciplinary analysis tool. Nucl. Instrum. Methods Phys. Res. B 181, 83–86 10.1016/S0168-583X(01)00564-X

[B17] Kim S. A. Punshon T. Lanzirotti A. Li A. Alonso J. M. Ecker J. R. (2006). Localization of iron in Arabidopsis seed requires the vacuolar membrane transporter VIT1. Science 314, 1295–1298 10.1126/science.1132563 17082420

[B18] Kraemer U. Clemens S. (2006). Functions and homeostasis of zinc, copper, and nickel in plants. Top. Curr. Genet. 14, 216–271 10.1007/4735_96

[B19] Lanquar V. Lelievre F. Bolte S. Hames C. Alcon C. Neumann D. (2005). Mobilization of vacuolar iron by AtNRAMP3 and AtNRAMP4 is essential for seed germination on low iron. EMBO J. 24, 4041–4051 10.1038/sj.emboj.7600864 16270029 PMC1356305

[B20] Laurette J. Larue C. Mariet C. Brisset F. Khodja H. Bourguignon J. (2012). Influence of uranium speciation on its accumulation and translocation in three plant species: oilseed rape, sunflower and wheat. Environ. Exp. Bot. 77, 96–107 10.1016/j.envexpbot.2011.11.007

[B20a] Lobinski R. Moulin C. Ortega R. (2006). Imaging and speciation of trace elements in biological environment. Biochimie 88, 1591–1604 10.1016/j.biochi.2006.10.003 17064836

[B21] Malan H. L. Mesjasz-Przybylowicz J. Przybylowicz W. J. Farrant J. M. Linder P. W. (2012). Distribution patterns of the metal pollutants Cd and Ni in soybean seeds. Nucl. Instrum. Methods Phys. Res. B. 273, 157–160 10.1016/j.nimb.2011.07.064

[B22] Marschner H. (2012). Mineral Nutrition of Higher Plants. San Diego, CA: Academic Press

[B23] Mayer M. (1999). SIMNRA, a simulation program for the analysis of NRA, RBS and ERDA. AIP Conf. Proc. 475, 541–544 10.1063/1.59188

[B24] Mesjasz-Przybylowicz J. Przybylowicz W. J. (2002). Micro-PIXE in plant sciences: present status and perspectives. Nucl. Instrum. Methods Phys. Res. B 189, 470–481 10.1016/S0168-583X(01)01127-2

[B25] Mikus M. Bobak M. Lux A. (1992). Structure of protein bodies and elemental composition of phytin from dry germ of maize (*Zea-Mays L*). Botanica Acta 105, 26–33 10.1111/j.1399-3054.1997

[B26] Morrissey J. Guerinot M. L. (2009). Iron uptake and transport in plants: the good, the bad, and the ionome. Chem. Rev. 109, 4553–4567 10.1021/cr900112r 19754138 PMC2764373

[B27] Murgia I. Arosio P. Tarantino D. Soave C. (2012). Biofortification for combating ‘hidden hunger’ for iron. Trends Plant Sci. 17, 47–55 10.1016/j.tplants.2011.10.003 22093370

[B27a] Ortega R. Deves G. Carmona A. (2009). Bio-metals imaging and speciation in cells using proton and synchrotron radiation X-ray microspectroscopy. J. R. Soc. Interface 6, S649–S658 10.1098/rsif.2009.0166.focus 19605403 PMC2843977

[B28] Otegui M. S. Capp R. Staehelin L. A. (2002). Developing seeds of Arabidopsis store different minerals in two types of vacuoles and in the endoplasmic reticulum. Plant Cell 14, 1311–1327 10.1105/tpc.010486 12084829 PMC150782

[B29] Pich A. Manteuffel R. Hillmer S. Scholz G. Schmidt W. (2001). Fe homeostasis in plant cells: does nicotianamine play multiple roles in the regulation of cytoplasmic Fe concentration? Planta 213, 967–976 10.1007/s004250100573 11722133

[B29a] Punshon T. Guerinot M. L. Lanzirotti A. (2009). Using synchrotron X-ray fluorescence microprobes in the study of metal homeostasis in plants. Ann. Bot. 103, 665–672 10.1093/aob/mcn264 19182222 PMC2707871

[B29b] Punshon T. Hirschi K. Yang J. Lanzirotti A. Lai B. Guerinot M. L. (2012). The role of CAX1 and CAX3 in elemental distribution and abundance in Arabidopsis seed. Plant Physiol. 158, 352–362 10.1104/pp.111.184812 22086421 PMC3252103

[B30] Ravet K. Touraine B. Boucherez J. Briat J. F. Gaymard F. Cellier F. (2009a). Ferritins control interaction between iron homeostasis and oxidative stress in Arabidopsis. Plant J. 57, 400–412 10.1111/j.1365-313X.2008.03698.x 18826427

[B31] Ravet K. Touraine B. Kim S. A. Cellier F. Thomine S. Guerinot M. L. (2009b). Post-translational regulation of AtFER2 ferritin in response to intracellular iron trafficking during fruit development in Arabidopsis. Mol. Plant 2, 1095–1106 10.1093/mp/ssp041 19825683

[B32] Roschzttardtz H. Conejero G. Curie C. Mari S. (2009). Identification of the endodermal vacuole as the iron storage compartment in the Arabidopsis embryo. Plant Physiol. 151, 1329–1338 10.1104/pp.109.144444 19726572 PMC2773051

[B33] Sarret G. Smits E. A. H. Michel H. C. Isaure M. P. Zhao F. J. Tappero R. (2013). Advances in Agronomy. San Diego, CA: Academic Press

[B34] Sarret G. Willems G. Isaure M. P. Marcus M. A. Fakra S. C. Frerot H. (2009). Zinc distribution and speciation in *Arabidopsis halleri* x *Arabidopsis lyrata* progenies presenting various zinc accumulation capacities. New Phytol. 184, 581–595 10.1111/j.1469-8137.2009.02996.x 19761446

[B35] Schneider T. Strasser O. Gierth M. Scheloske S. Povh B. (2002). Micro-PIXE investigations of apoplastic iron in freeze-dried root cross-sections of soil grown barley. Nucl. Instrum. Methods Phys. Res. B 189, 487–493 10.1016/S0168-583X(01)01129-6

[B36] Takahashi M. Nozoye T. Kitajima N. Fukuda N. Hokura A. Terada Y. (2009). *In vivo* analysis of metal distribution and expression of metal transporters in rice seed during germination process by microarray and X-ray Fluorescence Imaging of Fe, Zn, Mn, and Cu. Plant Soil 325, 39–51 10.1007/s11104-009-0045-7

[B37] Tylko G. Mesjasz-Przybylowicz J. Przybylowicz W. J. (2007). In-vacuum micro-PIXE analysis of biological specimens in frozen–hydrated state. Nucl. Instrum. Methods Phys. Res. B 260, 141–148 10.1016/j.nimb.2007.02.017

[B38] Vaughan J. G. Whitehouse J. M. (1971). Seed structure and the taxonomy of the Cruciferae. Bot. J. Linn. Soc. 64, 383–409 10.1111/j.1095-8339.1971.tb02153.x

[B39] Vogel-Mikus K. Pelicon P. Vavpetic P. Kreft I. Regvar M. (2009). Elemental analysis of edible grains by micro-PIXE: common buckwheat case study. Nucl. Instrum. Methods Phys. Rev. B 267, 2884–2889 10.1016/j.nimb.2009.06.104

[B40] Vogel-Mikus K. Pongrac P. Kump P. Necemer M. Simcic J. Pelicon P. (2007). Localisation and quantification of elements within seeds of Cd/Zn hyperaccumulator *Thlaspi praecox* by micro-PIXE. Environ. Pollut. 147, 50–59 10.1016/j.envpol.2006.08.026 17070633

[B41] Vogel-Mikus K. Regvar M. Mesjasz-Przybylowicz J. Przybylowicz W. J. Simcic J. Pelicon P. (2008). Spatial distribution of cadmium in leaves of metal hyperaccumulating *Thlaspi praecox* using micro-PIXE. New Phytol. 179, 712–721 10.1111/j.1469-8137.2008.02519.x 18554265

[B42] Young L. W. Westcott N. D. Attenkofer K. Reaney M. J. T. (2006). A high-throughput determination of metal concentrations in whole intact *Arabidopsis thaliana* seeds using synchrotron-based X-ray fluorescence spectroscopy. J. Synchrotron. Radiat. 13, 304–313 10.1107/S0909049506019571 16799221

